# Helicobacter Pylori Associated Antral Gastritis in Peptic Ulcer Disease Patients and Normal Healthy Population of Kashmir, India

**DOI:** 10.1155/DTE.4.135

**Published:** 1998

**Authors:** Gh. Jeelani Romshoo, G. M. Malik, M. Youssuf Bhat, Ab. Rashid rather, Javaid Ahmad Basu, Khursheed Ahmad Qureshi

**Affiliations:** 1 Department of Medicine (G.I.T. Division) SMHS Hospital Srinagar Kashmir 190 001 India; 2 Post Box No. 757 G.P.O. Srinagar Kashmir 190 001 India

## Abstract

*Aim*: To study the association of Helicobacter pylori infection with chronic antral gastritis in peptic ulcer disease patients and healthy population of Kashmir.

*Methods*: 50 peptic ulcer patients (duodenal ulcer = 46, gastric ulcer = 2 and combined duodenal and gastric ulcer = 2) and 30 asymptomatic healthy volunteers were included in this study. Peptic ulcer was diagnosed on endoscopic examination. 4–6 punch biopsies were taken from gastric antrum in all the individuals and in case of gastric ulcer an additional biopsy was taken from the edge of the ulcer to exclude its malignant nature. Helicobacter pylori (H. pylori) organism was diagnosed using three different test methods, viz. Histology (using Giemsa Stain), Microbiology (Gram Stain) and Biochemistry (using one minute Endoscopy Room Test). Histological diagnosis of H. pylori was taken as the “gold standard” for the presence of H. pylori organism. Histological diagnosis of gastritis was made using Hematoxylin and Eosin Stain and the gastritis was classified as active chronic gastritis and superficial chronic gastritis.

*Results*: Out of 30 peptic ulcer disease patients with associated antral gastritis, 27 (90%) were positive for H. pylori on histological examination (13 superficial chronic gastritis and 14 active chronic gastritis) whereas out of 8 healthy volunteers with histological evidence of chronic antral gastritis, H. pylori was observed in 7 individuals (87.50%) (4 active chronic gastritis and 3 superficial chronic gastritis).

*Conclusion*: A highly significant association between H. pylori infection with chronic antral gastritis both in peptic ulcer disease patients and healthy volunteers of Kashmir was found in this study. Association between H. pylori infection and chronic gastritis was 90% in peptic ulcer group and 87.50% in healthy population (P<0.005).


*Diagnostic and Therapeutic Endoscopy*, Vol. 4, pp. 135–139
Reprints available directly from the publisher
Photocopying permitted by license only

© 1998 OPA (Overseas Publishers Association)
Amsterdam B.V. Published under license
under the Harwood Academic Publishers imprint,
part of The Gordon and Breach Publishing Group.
Printed in Singapore.

### Helicobacter Pylori Associated Antral Gastritis in Peptic Ulcer Disease Patients and Normal Healthy Population of Kashmir, India

GH. JEELANI ROMSHOO*, G.M. MALIK, M. YOUSSUF BHAT, AB. RASHID RATHER, JAVAID AHMAD BASU and KHURSHEED AHMAD QURESHI

*Department of Medicine (G.I.T. Division), SMHS Hospital, Srinagar, Kashmir 190 001, India*

*(Received 27 March 1997; Revised 23 June 1997; In final form 25 July 1997)*

***Aim***: **To study the association of Helicobacter pylori infection with chronic antral gastritis in peptic ulcer disease patients and healthy population of Kashmir.**

***Methods***: **50 peptic ulcer patients (duodenal ulcer = 46, gastric ulcer = 2 and combined duodenal and gastric ulcer = 2) and 30 asymptomatic healthy volunteers were included in this study. Peptic ulcer was diagnosed on endoscopic examination. 4–6 punch biopsies were taken from gastric antrum in all the individuals and in case of gastric ulcer an additional biopsy was taken from the edge of the ulcer to exclude its malignant nature. Helicobacter pylori (H. pylori) organism was diagnosed using three different test methods, viz. Histology (using Giemsa Stain), Microbiology (Gram Stain) and Biochemistry (using one minute Endoscopy Room Test). Histological diagnosis of H. pylori was taken as the "gold standard" for the presence of H. pylori organism. Histological diagnosis of gastritis was made using Hematoxylin and Eosin Stain and the gastritis was classified as active chronic gastritis and superficial chronic gastritis.**

***Results***: **Out of 30 peptic ulcer disease patients with associated antral gastritis, 27 (90%) were positive for H. pylori on histological examination (13 superficial chronic gastritis and 14 active chronic gastritis) whereas out of 8 healthy volunteers with histological evidence of chronic antral gastritis, H. pylori was observed in 7 individuals (87.50%) (4 active chronic gastritis and 3 superficial chronic gastritis).**

***Conclusion***: **A highly significant association between H. pylori infection with chronic antral gastritis both in peptic ulcer disease patients and healthy volunteers of Kashmir was found in this study. Association between H. pylori infection and chronic gastritis was 90% in peptic ulcer group and 87.50% in healthy population ($P < 0.005$).**

*Keywords:* Active chronic gastritis, Duodenal ulcer, Gastric ulcer, Giemsa Stain, Hematoxylin and Eosin Stain, Healthy volunteers, Helicobacter pylori, Superficial chronic gastritis

* Corresponding author. Post Box No. 757, G.P.O. Srinagar, Kashmir 190 001, India.

### INTRODUCTION

Peptic ulcer disease (PUD) and chronic gastritis are most common disorders throughout the whole world. Chronic antral gastritis is a common finding in PUD especially in duodenal ulcer (DU). Chronic antral gastritis also occurs in gastric ulcers. One study has reported association between chronic gastritis and active duodenal ulcer as 100% compared to 50% in non-ulcer controls [1]. Since the isolation of small 'S' shaped, gramnegative, catalase and oxidase positive microaerophilic multiflagellate H. pylori from gastric antrum by Marshall *et al.* [2], a close relationship between chronic gastritis and H. pylori infection has been reported throughout the whole world. About 75% of patients with chronic gastritis have been found to have H. pylori infection compared to 10% in those without gastritis [2,3]. Helicobacter pylori infection has been strongly found to be associated with type B (chronic non-autoimmune) gastritis [2–4]. This type of chronic gastritis is associated with inflammation and patchy gastric atrophy. However, a preliminary study of dyspeptic patients from Bombay (India) found a non-significant association of 30% between antral Helicobacter pylori infection and chronic gastritis. Type A chronic gastritis thought to be of autoimmune etiology is rarely associated with H. pylori infection. Also a strong association has been found between the number of antral H. pylori organisms and severity of chronic gastritis [4–7].

A definite pathological association between H. pylori infection and gastritis is supported by (i) a positive correlation between chronic gastritis and H. pylori infection, (ii) a significant correlation between the number of H. pylori organism and the degree of polymorphonuclear (PMN) leukocyte infiltration in gastric mucosa, (iii) absent or decreased number of PMN infiltration in portions of gastric mucosa not associated with H. pylori infection, (iv) an improvement of chronic gastritis following eradication of H. pylori and (v) development of gastritis in healthy volunteers (in whom pre-ingestion antral biopsies were normal) following ingestion of H. pylori [8,9].

### MATERIAL AND METHODS

This study consisted of 50 PUD patients (DU = 46, GU = 2 and combined DU and GU = 2) whose disease was diagnosed on endoscopic examination and 30 asymptomatic healthy volunteers. Individuals with history of ingestion of steroids, non-steroidal anti-inflammatory drugs, antibiotics, bismuth preparations or metronidazole 4–6 weeks prior were not included in this study. The study was explained to the eligible individuals and informed written consent was obtained from each participant. The endoscopy was performed in the Gastroenterology section of SMHS Hospital of Government Medical College Srinagar, Kashmir. 4–6 antral biopsies were taken from each participant using sterilized biopsy forceps. One biopsy was used for one minute endoscopy room test, (Urease test) one for microbiological study (Gram's Stain) and another two for histological examination (For H. pylori and status of gastritis). An additional biopsy was taken from the edge of gastric ulcers to exclude its malignant nature.

### Histopathology

For histopathological study, paraffin sections of 3–5 μm thickness were taken of the biopsy material and these were stained with Hematoxylin and Eosin Stain (to study the histology of gastric antrum) and Giemsa Stain (For identification of H. pylori organism) separately. The Hematoxylin and Eosin stained tissue were broadly classified as:

(i) normal (when no inflammatory cells were seen),
(ii) active chronic gastritis (when PMN cell infiltration was observed in mucosal glands, pits or scattered throughout the epithelium or lamina propria and with intact mucosal surface) and

(iii) chronic superficial gastritis when inflammatory changes were confined to the lamina propria of the superficial mucosa and glands being preserved).

Detection of H. pylori on Giemsa Stain was taken as "Gold Standard" for the presence of H. pylori infection.

### One Minute Endoscopy Room Test (Urease Test)

A freshly prepared 10% W/V urea solution in deionized water at a PH of 6.8 was taken in two 5 ml capacity glass test tubes. Two drops of freshly prepared one percent phenol was added to each tube. One test tube served as a reagent and other as a control. One of the antral biopsy material was put into the reagent test tube and change of color from yellow to pink within 5 min was considered as positive thereby meaning the presence of H. pylori.

### Microbiology

One of the biopsy material was rubbed over a clean and dry glass slide and was heat fixed. The tissue was stained with Gram's Stain and observed under light microscope for the presence of gram negative, spiral shaped H. pylori organisms.

### Statistical Analysis

Chi-square test was used to analyze the results and a *P* value of less than 0.05 was taken to be significant.

### Ethics

Both the patients and volunteers were fully explained this study and informed consent was obtained from every participant for performing endoscopy and for obtaining biopsy tissue. Human experimentation guidelines laid by "Declaration of Helisinki" were followed. This study was approved by principal/Dean, Government Medical College, Srinagar, after consideration and approval by members of board of studies.

### RESULTS

Of the 50 peptic ulcer disease patients (DU = 46, GU = 2 and combined DU and GU = 2), 42 were males and 8 females, whereas out of 30 healthy volunteers 25 were males and five females in the mean age group of 30.88 ± 12.80 and 29.80 ± 11.22 respectively (Table I).

Out of 50 peptic ulcer disease (PUD) patients, 38 (76%) were positive for H. pylori on Giemsa Staining whereas out of 30 healthy volunteers, 10 (33.33%) were positive for H. pylori (Table II).

In PUD group, 30 (60%) were having antral gastritis on Hematoxylin and Eosin staining (14 with active chronic gastritis and 16 with superficial

TABLE I Characteristics of peptic ulcer disease patients and healthy volunteers

| | Peptic ulcer patients | Healthy volunteers |
|---|---|---|
| Number | 50 | 30 |
| Men/women | 42/8 | 25/5 |
| Age mean ± S.D. | 30.88 ± 12.80 | 29.80 ± 11.22 |

TABLE II Helicobacter pylori positivity among peptic ulcer patients and healthy volunteers by different test methods

| Group | Total number | H. Pylori positivity | | |
|---|---|---|---|---|
| | | Histology | OMERT (one minute endoscopy room test) | Gram Stain |
| PUD patients | 50 | 38 (76%) | 40 (80%) | 37 (74%) |
| Controls | 30 | 10 (33.33%) | 10 (33.33%) | 9 (30%) |

TABLE III Helicobacter pylori positivity by different test methods in relation to type of antral gastritis (in peptic ulcer patients)

| Total number studied | Total No. with gastritis (on histology) | Type of gastritis and number | H. pylori positivity | | |
|---|---|---|---|---|---|
| | | | Histology No. (%) | Gram's Stain No. (%) | *OMERT No. (%) |
| 50 | 30 | Active chronic gastritis (14) | 13 (92.86) | 11 (78.57) | 13 (92.86) |
| | | Superficial chronic gastritis (16) | 14 (87.50) | 13 (81.25) | 12 (75.00) |

*OMERT = One Minute Endoscopy Room Test.

TABLE IV Helicobacter pylori positivity by different test methods in relation to type of antral gastritis (in healthy volunteers)

| Total number studied | Total number with antral gastritis (on histology) | Type of gastritis and their number | H. pylori positivity | | |
|---|---|---|---|---|---|
| | | | Histology No. (%) | Gram's Stain No. (%) | *OMERT No. (%) |
| 30 | 8 | Active chronic gastritis (4) | 4 (100) | 2 (50) | 4 (100) |
| | | Superficial chronic gastritis (4) | 3 (75) | 2 (50) | 3 (75) |

*OMERT = One Minute Endoscopy Room Test.

TABLE V Correlation between H. pylori status by histology and antral gastritis in peptic ulcer patients and healthy volunteers

| Group | Number | Gastritis present H. pylori status | | Gastritis absent H. pylori status | |
|---|---|---|---|---|---|
| | | Present | Absent | Present | Absent |
| Peptic ulcer patients | 50 | 27 | 3 | 8 | 12 |
| Healthy volunteers | 30 | 7 | 1 | 3 | 19 |
| Total | 80 | 34 | 4 | 11 | 31 |

Probability values determined by the $x^2$ test with yates correction demonstrated that the differences between the presence or absence of chronic gastritis in relation to presence or absence of H. pylori by histology were highly significant ($P < 0.002$ to $P < 0.00005$).

chronic gastritis). 92.86% of active chronic gastritis and 87.50% of superficial chronic gastritis were positive for H. pylori organism on Giemsa Staining (Table III) whereas in healthy volunteers group, 8 (26.67%) had histological evidence of chronic gastritis (4 having active chronic gastritis and 4 superficial chronic gastritis). All the four volunteers having active chronic gastritis were positive for H. pylori organism (100% positivity) whereas three volunteers with superficial chronic gastritis (75%) were positive for H. pylori organism on Giemsa Staining (Table IV). A highly significant ($P < 0.002$ to $P < 0.00005$) was found between H. pylori positivity on histology and chronic active gastritis (Table V).

### DISCUSSION

Helicobacter pylori organisms has been found to play an important pathogenic role in peptic ulcer disease (especially in duodenal ulcer), gastritis and recently in gastric cancer [2,4,6,7]. The antral colonization with H. pylori organism has been reported from every corner of the globe especially from developing and underdeveloped countries [2,4,6]. This study was carried out in a highly endemic peptic ulcer disease area of India [10] to find out any association between chronic gastritis and Helicobacter pylori positivity among peptic ulcer patients and asymptomatic healthy volunteers.

In this study the association of Helicobacter pylori with peptic ulcer disease (duodenal ulcer) was found to be 76% and prevalence of H. pylori in healthy asymptomatic volunteers was found to be 33.33%. The association with peptic ulcer disease was statistically significant ($P < 0.005$). The association between H. pylori positivity and chronic antral gastritis was also very significant in both peptic ulcer disease and healthy volunteers ($P < 0.002$ to $P < 0.00005$). Despite absence of symptoms or risk features 26.67% of healthy volunteers were found to have histologic antral gastritis and 87.50% of these were positive for H. pylori organism. Thereby showing direct relation between chronic gastritis and H. pylori positivity.

This study confirms the data published from different parts of the world [2–8] but contradicts the earlier study published from India [11], in which study on 95 dyspeptic patients found a non-significant association of 30% between antral Helicobacter pylori infection and chronic gastritis.

In conclusion, this study found a highly significant association between Helicobacter pylori infection and peptic ulcer disease (duodenal ulcer). Also a very significant association between H. pylori infection and chronic antral gastritis was found in both peptic ulcer group and asymptomatic healthy volunteers.

### *References*

[1] Hui, W.M., Lam, S.K., Ho, J., Matthew, M.N., Lui, I., Lai, C.I., Lok, A.S., Lau, W.Y., Poon, G.P. *et al.* Chronic antral gastritis in duodenal ulcer. Natural history and treatment with prostaglandin E. *Gastroenterology* 1986; **91**: 1095–1101.

[2] Marshall, B.J. and Warren, J.R. Unidentified curved bacilli in the stomach of patients with gastritis and peptic ulceration. *Lancet* 1984; **1**: 1311–5.

[3] Rauws, E.A.J., Langenberg, W., Hendrik, J.H., Zanen, H.C. and Tytgat, G.N.J. Campylobacter pyloridis-associated chronic active antral gastritis: A prospective study of its prevalence and the effects of antibacterial and anti ulcer treatment. *Gastroenterology* 1988; **94**: 33–40.

[4] Buck, G.E., Gourley, W.K., Lee, W.K. *et al.* Relation of campylobacter pyloridis to gastritis and peptic ulcer. *J. Infect. Dis.* 1986; **152**: 664–9.

[5] Bayerdorffer, E., Oertrel, H., Lehn, N. *et al.* Topographic association between active gastritis and campylobacter pylori colonisation. *J. Clin. Pathol.* 1989; **42**: 834–9.

[6] Goodwin, C.S., Armstrong, J.A. and Marshall, B.J. Campylobacter pyloridis, gastritis and peptic ulceration. *J. Clin. Pathol.* 1986; **39**: 353–65.

[7] Mc Nulty, C.M.A. and Watson, D.M. Spiral Bacteria of the gastric antrum. *Lancet* 1984; **1**: 1068–9.

[8] Marshall, B.J., Armstrong, J.A., McGechie, D.B. and Glancy, R.J. Attempt to fulfil Koch's postulates for pyloric campylobacter. *Med. J. Aust.* 1985; **142**: 436–9.

[9] Marshall, B.J., Hislop, I., Glancy, R. and Armstrong, J. HIstological improvement of active chronic gastritis in patients treated with De-nol. *Aust. N. A. J. Med.* 1984; **14**(Suppl. 4): 907.

[10] Durrani, H. Duodenal ulcer profile in a highly Endemic area. *JAPI* 1990; **38**(Suppl. I): 692–694.

[11] Nanivadekar, S.A., Sawant, P.D., Saraswathi, K., Shroff, C.P. *et al.* Association of campylobacter pylori with gastritis, duodenal ulcer and gastric ulcer – a preliminary report of dyspeptic patient. *Indian J. Gastroenterol.* 1988; **7**(3): 141–142.

